# Clinical and demographic characteristics of hospitalized patients with COVID-19 referred to a Consultation - Liaison Psychiatry Unit

**DOI:** 10.1192/j.eurpsy.2023.487

**Published:** 2023-07-19

**Authors:** A. Papadopoulou, V. Efstathiou, E. Koliou, K. Papazachos, A. Barbari, N. Kollia, A. Karvouni, I. Theodoridou, E. Rizos, N. Smyrnis

**Affiliations:** ^1^Second Department of Psychiatry, National and Kapodistrian University of Athens, “Attikon” University General Hospital; ^2^Psychology Department, National and Kapodistrian University of Athens, Athens, Greece

## Abstract

**Introduction:**

There is accumulating evidence that SARS-CoV-2 infection, apart from physical complications, can cause a variety of symptoms related to mental health, either during the acute phase of the infection or following the resolution of acute COVID-19 (i.e., long-COVID).

**Objectives:**

To investigate the demographic and clinical characteristics of a sample of hospitalized patients with COVID-19.

**Methods:**

Data were collected from 1 January 2021 to 31 May 2022. In particular, clinical and demographic characteristics of patients hospitalized with COVID-19 at the “Attikon” University General Hospital and who were referred for assessment to the Consultation Liaison Psychiatry unit were collected and analyzed.

**Results:**

During the study period, 107 patients, 66 men (62%) and 41 women (38%) with a mean age of 63 years, with COVID-19 were referred to the Consultation Liaison Psychiatry unit for evaluation. Among them, 58 (54.6%) had a previous psychiatric history, while 49 (45.4%) were assessed for the first time by a mental health professional. The most frequent psychiatric manifestations included anxiety manifestations [38 patients (36%)], delirium [37 patients (35%)] and depressive manifestations [15 patients (14%)].

**Conclusions:**

The description of demographic and clinical characteristics of hospitalized COVID-19 patients with concurrent psychiatric manifestations highlights the importance of early clinical detection of psychiatric comorbidity by physicians with a view to ensuring that patients’ needs are supported in an integrated, holistic and patient-centric manner.

**Disclosure of Interest:**

None Declared

